# Deep Learning for Reaction-Diffusion Glioma Growth Modeling: Towards a Fully Personalized Model?

**DOI:** 10.3390/cancers14102530

**Published:** 2022-05-20

**Authors:** Corentin Martens, Antonin Rovai, Daniele Bonatto, Thierry Metens, Olivier Debeir, Christine Decaestecker, Serge Goldman, Gaetan Van Simaeys

**Affiliations:** 1 Department of Nuclear Medicine, Hôpital Erasme, Université libre de Bruxelles, Route de Lennik 808, 1070 Brussels, Belgium; antonin.rovai@erasme.ulb.ac.be (A.R.); serge.goldman@ulb.be (S.G.); gaetan.vansimaeys@ulb.be (G.V.S.); 2 Center for Microscopy and Molecular Imaging (CMMI), Université libre de Bruxelles, Rue Adrienne Bolland 8, 6041 Charleroi, Belgium; olivier.debeir@ulb.be (O.D.); christine.decaestecker@ulb.be (C.D.); 3 Laboratory of Image Synthesis and Analysis (LISA), École Polytechnique de Bruxelles, Université libre de Bruxelles, Avenue Franklin Roosevelt 50, 1050 Brussels, Belgium; daniele.bonatto@ulb.be (D.B.); thierry.metens@ulb.be (T.M.); 4 Department of Radiology, Hôpital Erasme, Université libre de Bruxelles, Route de Lennik 808, 1070 Brussels, Belgium

**Keywords:** cellularity, deep convolutional neural network, glioma, magnetic resonance imaging, reaction-diffusion model, tumor growth modeling

## Abstract

**Simple Summary:**

Mathematical tumor growth models have been proposed for decades to capture the growth of gliomas, an aggressive form of brain tumor. However, the estimation of the tumor cell-density distribution at diagnosis and model parameters from partial observations provided by magnetic resonance imaging are ill-posed problems. In this work, we propose a deep learning-based approach to address these problems. 1200 synthetic tumors are first generated using the mathematical model over brain geometries of 6 volunteers. Two deep convolutional neural networks are then trained to (i) reconstruct a whole tumor cell-density distribution and (ii) evaluate the model parameters from partial observations provided in the form of threshold-like imaging contours, with state-of-the-art results. From the estimated cell-density distribution and parameter values, the spatio-temporal evolution of the tumor can ultimately be accurately captured by the mathematical model. Such an approach could be of great interest for glioma characterization and therapy planning.

**Abstract:**

Reaction-diffusion models have been proposed for decades to capture the growth of gliomas, the most common primary brain tumors. However, ill-posedness of the initialization at diagnosis time and parameter estimation of such models have restrained their clinical use as a personalized predictive tool. In this work, we investigate the ability of deep convolutional neural networks (DCNNs) to address commonly encountered pitfalls in the field. Based on 1200 synthetic tumors grown over real brain geometries derived from magnetic resonance (MR) data of six healthy subjects, we demonstrate the ability of DCNNs to reconstruct a whole tumor cell-density distribution from only two imaging contours at a single time point. With an additional imaging contour extracted at a prior time point, we also demonstrate the ability of DCNNs to accurately estimate the individual diffusivity and proliferation parameters of the model. From this knowledge, the spatio-temporal evolution of the tumor cell-density distribution at later time points can ultimately be precisely captured using the model. We finally show the applicability of our approach to MR data of a real glioblastoma patient. This approach may open the perspective of a clinical application of reaction-diffusion growth models for tumor prognosis and treatment planning.

## 1. Introduction

Gliomas are the most common primary brain tumors, and remain associated with a poor prognosis. Among them, diffuse gliomas are known to be highly infiltrative tumors in which invading tumor cells can be found as far as 4 cm from the gross tumor [1]. However, the limited sensibility of magnetic resonance imaging (MRI)—the modality of choice for glioma imaging—to changes occurring at the cellular level makes the delineation of the whole tumor extent tedious and often leads to sub-optimal treatment plannings.

Knowledge of the whole tumor cell distribution, within and beyond the outlines of the tumor visible on MRI, would instead allow us to refine surgery or radiation therapy planning. Current standards for the planning of such therapies indeed rely on the addition of a fixed margin to account for tumor infiltration, defining the clinical target volume [2,3]. From an estimated tumor cell distribution, dose deposition could instead be redistributed, with a higher dose delivered to areas more likely to contain tumor cells and, on the other hand, a lower dose delivered to surrounding healthy tissues, while keeping the total dose unchanged [2]. Furthermore, evaluation of the tumor growth dynamics from repeated medical imaging data would also be of great interest to better characterize the tumor, anticipate its growth, and identify its probable migration pathways as well as areas prone to recurrence.

Reaction-diffusion tumor growth models have been studied for decades to circumvent the limitations of current medical imaging techniques and improve treatment planning in gliomas [2,4,5,6,7,8,9,10]. These models rely on partial differential equations (PDEs) to capture the spatio-temporal evolution of a tumor cell-density function over the brain domain, driven by tumor cell migration and proliferation. The most commonly used form involves a logistic reaction term and is referred to as the Fisher equation [11]:

(1) $$\frac{\partial c(x,t)}{\partial t}=d\;\nabla^{2}c\left(x,t\right)+\rho \;c\left(x,t\right)\left(1-c(x,t)\right)$$

where c(x,t) is the tumor cell-density at location x and time *t* normalized by the maximum carrying capacity $c_{\max}$ of brain tissues (i.e., c(x,t)∈[0,1],∀x,t), *d* is the tumor cell diffusion coefficient, and ρ is the tumor cell proliferation rate. A property of the well-studied Equation (1) is that, under certain conditions and for constant coefficients, it admits a traveling wave solution on the infinite cylinder with propagation speed $v=2\sqrt{d\;\rho }$, whose profile decays exponentially with decay constant $\lambda =\sqrt{d/\rho }$ as the distance to the origin tends to infinity and for large but finite times *t* [10,12].

Since their first introduction by Murray and colleagues in the early 1990s [4], reaction-diffusion growth models have been continuously improved to successively integrate (i) a variable tumor cell diffusion rate in white versus gray matter [13] and (ii) an anisotropic diffusion tensor field accounting for the preferred migration of tumor cells along white matter tracts, whose orientation can be assessed by diffusion tensor imaging (DTI) [14]. These improvements led to the formulation that is used throughout this work, presented in Section 2.1. Tumor-induced mass effect [6,7], necrosis, and hypoxia [15,16], as well as the effects of surgery [5], chemotherapy [4,17,18], and radiotherapy [18,19] have also been introduced into reaction-diffusion glioma growth models, but are not considered in this work. For a more detailed overview of reaction-diffusion glioma growth modeling, the reader is referred to [2,4,5,6,7,8,9,10].

Although reaction-diffusion models have shown promising results for patient follow-up and improved radiotherapy planning [2], their clinical application is still prone to severe limitations. Indeed, the estimation of the parameter values and the tumor cell-density distribution at diagnosis time—required to predict the tumor evolution at later times—but also the validation of such models in vivo, implies to extract information on the tumor at the cellular level from medical imaging data. To address this issue, Swanson and colleagues have proposed in [8] to model the imaging function of MRI sequences $I_{\mathrm{seq}}\left(x,t\right)$—indicating whether a tumor-induced abnormality is visible at location x and time *t* on the image—as a simple tumor cell-density threshold function:

(2) $$I_{\mathrm{seq}}\left(x,t\right)=\left\{\begin{array}{cc} 1\; & \mathrm{if}\;c\left(x,t\right)\geq c_{\mathrm{seq}}\\ 0\; & \mathrm{otherwise} \end{array}\right.$$

where $c_{\mathrm{seq}}$ is the tumor cell-density detectability threshold of the sequence. The abnormalities considered in [8] were the hyper-intense enhancing tumor core visible on T1-weighted sequences with injection of gadolinium-based contrast agent (T1Gd) and the peritumor vasogenic edema visible on T2-weighted sequences with or without fluid-attenuated inversion-recovery (T2/T2 FLAIR). Based on these assumptions, the authors suggested that the outlines of these abnormalities would correspond to iso-contours of the tumor cell-density function—i.e., hyper-surfaces along which *c* has a constant value:

(3) $$\begin{array}{c} c\left(x,t\right)=c_{\mathrm{seq}},\;\forall x\in \partial \Omega_{\mathrm{abn}} \end{array}$$

where $\partial \Omega_{\mathrm{abn}}$ is the boundary of the visible abnormality.

Building upon this work, Konukoglu and colleagues proposed in [10] a fast marching approach to construct an approximate solution of Equation (1) at imaging time which satisfies Equation (3). This approach has the interesting property of not attempting to dynamically solve the model but seeks to extrapolate the tumor invasion beyond its MR-visible margins within the reaction-diffusion framework, with applications for radiotherapy planning. It has, nevertheless, two major limitations: First, it requires the ability to extract a tumor cell-density iso-contour from the image, from which the whole tumor cell distribution is built. However, we showed in a previous work based on histological data that the outlines of the edema visible on T2 FLAIR MR images do not coincide with a cell-density iso-contour [20]. The proposed explanation is that, due to spatial discontinuities of the tumor cell-density function at interfaces between white and gray matter as well as along the brain domain boundary, Equation (2) does not necessarily imply Equation (3). The second limitation of this approach is that the method still needs to specify the diffusivity and proliferation rate of the tumor, which are unknown at imaging time and need to be adjusted to each tumor.

The estimation of the model parameter values from medical imaging data has also been addressed previously. In [21], the definition of the asymptotic speed of the tumor front $v=2\sqrt{d\;\rho }$ is used to estimate the tumor cell diffusivity $d_{\mathrm{white}}$ and $d_{\mathrm{gray}}$ in white and gray matter using a fast marching approach. However, the method does not allow us to separate the individual contributions of *d* and ρ to *v*, hence ρ is supposed constant for all tumors. Furthermore, this estimation is only valid for large times for which the traveling-wave approximation holds. The approach was then further extended in [9] to take into account the transient speed evolution and the curvature of the tumor front, but still considers a constant ρ value for all tumors. Besides, these fast marching formulations make the assumption that the outlines of the peritumor vasogenic edema visible on T2 MR images correspond to an iso-contour of the traveling wave arrival time function. However, this hypothesis might not be verified due to discontinuities appearing at the brain boundary voxels, which could have been reached long before the imaging time. In [22], a Bayesian approach is used to estimate both the diffusion and proliferation parameters of the model from two imaging contours obtained by Equation (2) at two different times. The method was found to accurately estimate the infiltration length $\lambda =\sqrt{d/\rho }$ of the tumor, but less accurately the tumor front propagation speed $v=2\sqrt{d\;\rho }$, based on synthetic and real glioblastoma (GBM) MRI data. In [18], parameter values of a two-species reaction-diffusion model incorporating tumor-induced mass effect and response to chemoradiation are estimated based on tumor cell-density distributions derived from longitudinal T1Gd, T2 FLAIR, and diffusion-weighted (DW) MR data, with promising results. However, the cell-density distributions used to initialize the model and fit the parameters were built piecewise from the enhancing/non-enhancing tumor regions delineated on T1Gd/T2 FLAIR images as well as average diffusion coefficient (ADC) maps derived from DW-MR data, and are therefore not guaranteed to be solution of Equation (1) nor to reflect the actual tumor cell distribution.

Tumor source localization is another widely addressed problem in reaction-diffusion glioma growth modeling. In [23], an inverse problem approach is used to estimate the tumor source location from a given tumor cell-density distribution, with promising results. However, to be applicable in clinical practice, the method still requires the ability to derive a whole tumor cell-density distribution from medical imaging data.

Finally, several works have attempted to jointly estimate the tumor source location and model parameters from patient imaging data. In [24], a PDE-constrained optimization approach is used to assess the source location and parameter values of a reaction-diffusion glioma growth model including an additional advection term. The tumor growth model is coupled to a linear elastic model for the tumor-induced mass effect. Two optimization criteria are used in the study: (i) the squared difference between the true and estimated cell-density fields at given imaging times and (ii) the squared distance between the true and estimated position of manually defined landmarks on staggered scans, that are displaced as the surrounding brain tissues are deformed under mass effect. However, the first criterion requires the knowledge of the true tumor cell-density field, which again cannot be derived directly from imaging data. Promising results were obtained for the landmark-based criterion on a real glioma case but strong assumptions are made on the initial cell-density field—supposedly Gaussian—and no ground truth was available to assess the model parameter estimation. In [25], the fast marching approach of Konukoglu and colleagues [9,21] is used to assess the diffusivity ratio $d_{\mathrm{white}}/d_{\mathrm{gray}}$ along with the tumor source location, but a fixed proliferation rate ρ was again considered. More recently, a Bayesian framework has been proposed to simultaneously estimate the tumor source, emergence time, diffusivity, and proliferation rate from a combination of T1Gd, T2 FLAIR, and [^18^F]fluoroethyl-L-tyrosine ([^18^F]FET) positron emission tomography (PET) images in [26]. However, the study reported that these last three parameters cannot be individually assessed from a single imaging time point. Finally, in [27] a numerical approach is proposed to solve the ill-posed problem of estimating both the tumor initial location as well as its diffusivity and proliferation rate, and is further applied on 206 GBM cases from the BraTS dataset [28] in [29], for which actual parameter values are however not known. Although the method has shown promising results and only requires a single imaging time point, it still has three major limitations: (i) it only allows for the estimation of unscaled dimensionless diffusion and proliferation parameters as the time between tumor emergence and imaging is unknown, which restricts the absolute comparison of the estimated parameter values between tumors and prevents the use of the model as a personalized prediction tool for the tumor evolution over time, (ii) it makes strong assumptions regarding the initial tumor cell-density distribution—a sparse set of Gaussian distributions with identical standard deviation and a maximum density value of 1 over the set—and (iii) it relies on many user-defined parameters, to some of which the method is reported sensitive [27,29].

In addition to their various limitations highlighted hereabove, none of the aforementioned works have jointly addressed the estimation of the tumor cell-density distribution at diagnosis time and individual diffusion and proliferation parameters of the model, which could however made it possible to anticipate the growth of the tumor at further times using the model and thus adapt treatment strategies. Besides, most of these works considered a spatially constant diffusion coefficient in white matter and/or an identical proliferation rate for all tumors, which is not realistic. The introduction of an arbitrary diffusion tensor field D(x) and a tumor-specific proliferation rate ρ would make the addressed problems even more challenging.

Over the last five years, the advent of deep-learning techniques—and in particular deep convolutional neural networks (DCNNs)—has opened tremendous opportunities in the field of medical imaging, achieving state-of-the-art performance in many image classification and segmentation challenges [30]. One interesting property of deep neural networks is their ability to approximate any function under certain conditions [31]. This property makes the technique attractive for the problems addressed in this work. DCNNs may indeed be used to approximate solutions of PDEs such as Equation (1) over complex domains, and for spatially variable coefficients, as well as to estimate their parameter values from partial observations provided in the form of threshold-like imaging contours.

In this work, we investigate the ability of DCNNs to address common pitfalls encountered in the clinical application of reaction-diffusion glioma growth models. In particular, we focus on the following two tasks:

1. Reconstructing a whole brain tumor cell-density distribution compatible with the reaction-diffusion model from a pair of contours obtained through a threshold-like imaging process as in Equation (2) for two different detectability threshold values at a given imaging time. These contours may for example correspond to the outlines of the enhancing core and peritumor vasogenic edema on T1Gd and T2/T2 FLAIR MR images, respectively.
2. Estimating the values of the diffusion and proliferation parameters of the model from three imaging contours: (i) two contours obtained for a first detectability threshold value (e.g., the vasogenic edema outlines) at two different imaging times and (ii) a third contour obtained for a second detectability threshold value (e.g., the enhancing core outlines) at the second imaging time.

We demonstrate the ability of DCNNs to perform these tasks accurately based on 1200 synthetic tumors grown over brain geometries derived from the MR data of six healthy subjects. We also show the applicability of our approach on MR data of a real glioblastoma patient.

## 2. Materials and Methods

### 2.1. The Reaction-Diffusion Model

The reaction-diffusion tumor growth model that is used throughout this work is described by Equations (4)–(6) [6,25,32]:

$$\left\{\begin{array}{ccc} \frac{\partial c(x,t)}{\partial t}=\nabla \cdot (D(x)▽c(x,t))+\rho c(x,t)(1-c(x,t)) & \forall x\in \Omega ,\forall t>0 & \left(4\right)\\ c(x,0)=c_{0}(x) & \forall x\in \Omega & \left(5\right)\\ D(x)\nabla c(x,t)\cdot n_{\partial_{\Omega }}\left(x\right)=0 & \forall x\in \partial \Omega & \left(6\right) \end{array}\right.$$

where c(x,t) is the tumor cell-density at location x and time *t* normalized by the maximum carrying capacity $c_{\max}$ of brain tissues (i.e., c(x,t)∈[0,1],∀x,t), D(x) is the symmetric tumor cell diffusion tensor at location x, ρ is the tumor cell proliferation rate, $c_{0}\left(x\right)$ is the initial tumor cell-density at location x, and $n_{\partial_{\Omega }}\left(x\right)$ is a unit normal vector pointing outwards the boundary $\partial_{\Omega }$ of the brain domain Ω at location $x\in \partial_{\Omega }$. Equation (5) specifies the initial condition of the problem whereas Equation (6) provides no-flux Neumann boundary conditions reflecting the inability of tumor cells to diffuse across $\partial_{\Omega }$.

### 2.2. MR Data Acquisition

For the needs of this work, 6 healthy volunteers were enrolled for an MRI acquisition comprising a T1 BRAVO (echo time: ∼3 ms, repetition time: ∼8.3 ms, inversion time: 450 ms, flip angle: 12${}^{{}^{\circ}}$, pixel bandwidth: 244 Hz ${\mathrm{voxel}}^{-1}$, slice thickness/spacing: 1/1 mm, matrix: 240 voxel×240 voxel×172 voxel, field of view: 250 mm×175 mm×174 mm), a T2 FLAIR (echo time: ∼119 ms, repetition time: 7.2 s, inversion time: ∼2040 ms, flip angle: 90${}^{{}^{\circ}}$, bandwidth: 122 Hz ${\mathrm{voxel}}^{-1}$, phase/slice acceleration factor: 2/2, slice thickness/spacing: 1.4/0.7 mm, matrix: 256 voxel×256 voxel×252 voxel, field of view: 256 mm×245.8 mm×176.4 mm), and an EPI-DTI (echo time: 77.1 ms, repetition time: 7 s, inversion time: 108 ms, flip angle: 90${}^{{}^{\circ}}$, bandwidth: 1953.12 Hz ${\mathrm{voxel}}^{-1}$, phase/slice acceleration factor: 2/1, multiband factor: 3, slice thickness/spacing: 2/2 mm, matrix: 120 voxel×120 voxel, slices: 72, field of view: 240 mm×240 mm×144 mm, directions: 32, *b*-value: 1000 s ${\mathrm{mm}}^{-2}$) sequence. To correct for susceptibility-induced distortions (see Section 2.3.1), a second DTI acquisition with reversed phase-encode polarity and only 6 directions was additionally performed. All acquisitions were performed on a 3 T Signa PET/MR scanner (GE Healthcare, Chicago, IL, USA) with a Nova 32-channel head coil (Nova Medical, Houston, TX, USA).

To illustrate our approach (see Section 2.6), similar T1 BRAVO, T2 FLAIR, and DTI images as well as an additional T1Gd (echo time: 3.2 ms, repetition time: 8 ms, flip angle: 8${}^{{}^{\circ}}$, pixel bandwidth: 255 Hz ${\mathrm{voxel}}^{-1}$, slice thickness/spacing: 1/1 mm, matrix: 232 voxel×231 voxel×175 voxel, field of view: 230 mm×190.3 mm×144 mm) image acquired on a 3 T Achieva scanner (Philips Medical Systems, Eindhoven, The Netherlands) of a 69-year-old male patient with IDH-wildtype GBM were retrospectively analyzed.

### 2.3. Processing

#### 2.3.1. DTI Data Processing

The acquired DTI data were first corrected for susceptibility-induced distortion, eddy currents, and patient motion using the topup and eddy tools available as part of FSL [33]. A water diffusion tensor field $D_{\mathrm{water}}\left(x\right)$ was then reconstructed from the corrected DTI data by least-squares fitting using FSL’s dtifit tool. The whole FSL script used for DTI data processing is available in Appendix A.

#### 2.3.2. Resampling and Registration

The acquired T1 BRAVO, T1Gd (GBM patient only), and T2 FLAIR images as well as the corrected DTI data and the derived water diffusion tensor field were resampled to an isotropic voxel size of 1mm×1mm×1mm by linear interpolation. To correct for patient motion throughout the acquisition, the T1 BRAVO and T2 FLAIR images were rigidly registered to the b=0 DTI image used as reference by maximization of their mutual information [34]. All resampling and registration steps were performed using an in-house software in C++ based on ITK [35] and VTK [36].

#### 2.3.3. Skull Stripping

The brain volume was then segmented on the registered T2 FLAIR image using the Otsu thresholding [37] followed by a morphological erosion with structuring element of radius 2 voxel, a largest component extraction, a morphological dilation of radius 2 voxel, a morphological closing of radius 8 voxel, and a morphological dilation of radius 1 voxel.

#### 2.3.4. Brain Tissue Segmentation

**Figure 1:**
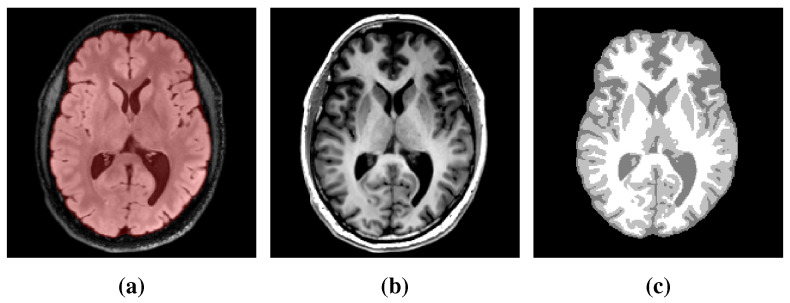
Example of processed MR data. (**a**) Axial slice of the T2 FLAIR image with superimposed segmented brain mask (red). (**b**) Corresponding slice of the T1 BRAVO image. (**c**) Segmented brain map obtained with the MICO algorithm [38] followed by manual corrections.

The extracted brain domain was further segmented on the registered T1 BRAVO image into white matter, gray matter, and cerebrospinal fluid using an in-house implementation of the MICO intensity-based clustering algorithm comprising a bias field correction step [38]. Manual corrections were further applied to the mis-segmented basal nuclei and falx cerebri. This last step is crucial to prevent the migration of tumor cells between brain hemispheres via routes other than the corpus callosum, as highlighted previously [2]. The segmentation results were finally merged into a single brain map. An example of T2 FLAIR and T1 BRAVO images with the corresponding brain mask and segmented brain map is depicted in Figure 1.

#### 2.3.5. Tumor Segmentation

For illustration purposes (see Section 2.6), the enhancing core and peritumor vasogenic edema were also semi-automatically segmented on the T1Gd and T2 FLAIR images of the GBM patient using combinations of thresholding, connected component extraction, and morphological operations.

#### 2.3.6. Tumor Diffusion Tensor

A tumor diffusion tensor field D(x) was built piecewise from the DTI-derived water diffusion tensor $D_{\mathrm{water}}\left(x\right)$ and the segmented brain domain Ω as follows:

(7) $$D\left(x\right)=\left\{\begin{array}{cc} D_{\mathrm{white}}\left(x\right)\; & \mathrm{if}\;x\in \Omega_{\mathrm{white}}\\ D_{\mathrm{gray}}\; & \mathrm{if}\;x\in \Omega_{\mathrm{gray}}\\ 0\; & \mathrm{otherwise} \end{array}\right.$$

where $D_{\mathrm{white}}\left(x\right)$ and $D_{\mathrm{gray}}$ are the tumor cell diffusion tensor fields within the white and gray matter domains $\Omega_{\mathrm{white}}$ and $\Omega_{\mathrm{gray}}$, respectively, and 0 is the null tensor, with:

(8) $$D_{\mathrm{gray}}=\left[\begin{array}{ccc} d_{\mathrm{gray}} & 0 & 0\\ 0 & d_{\mathrm{gray}} & 0\\ 0 & 0 & d_{\mathrm{gray}} \end{array}\right]$$

where $d_{\mathrm{gray}}$ is the mean diffusivity of tumor cells in gray matter.

The white matter tumor cell tensor field $D_{\mathrm{white}}\left(x\right)$ was built from the DTI-derived water diffusion tensor $D_{\mathrm{water}}\left(x\right)$ using the method proposed by Jbabdi and colleagues in [14]. This step is motivated by the observation that, for mechanical [39] and molecular [40] reasons, tumor cells preferentially migrate along rather than across brain fibers, similarly to diffusing water molecules. The method consists of increasing the degree of anisotropy of the water diffusion tensor and scaling its mean diffusivity $\mathrm{MD}=\mathrm{Tr}\left(D_{\mathrm{water}}\right)/3$ to account for the difference in diffusive behavior between tumor cells and water molecules, $\mathrm{Tr}\left(A\right)$ being the trace of matrix A [14]:

(9) $$D_{\mathrm{white}}\left(x\right)=\frac{3\;d_{\mathrm{white}}}{\sum_{i=1}^{3}{\tilde{\lambda }}_{i}\left(a\right)}\sum_{i=1}^{3}{\tilde{\lambda }}_{i}\left(a\right)\;e_{i}\left(x\right)\;e_{i}^{⊤}\left(x\right)$$

where $d_{\mathrm{white}}$ is the mean diffusivity of tumor cells in white matter, $e_{i}\left(x\right)$ is the *i*th eigenvector of $D_{\mathrm{water}}\left(x\right)$, and ${\tilde{\lambda }}_{i}\left(a\right)=l_{i}\left(a\right)\lambda_{i}$, with:

(10) $$\begin{array}{c} \left[\begin{array}{c} l_{1}\left(a\right)\\ l_{2}\left(a\right)\\ l_{3}\left(a\right) \end{array}\right]=\left[\begin{array}{ccc} a & a & 1\\ 1 & a & 1\\ 1 & 1 & 1 \end{array}\right]\left[\begin{array}{c} c_{l}\\ c_{p}\\ c_{s} \end{array}\right], \end{array}$$

(11) $$c_{l}=\frac{\lambda_{1}-\lambda_{2}}{\lambda_{1}+\lambda_{2}+\lambda_{3}},\;c_{p}=\frac{2\;(\lambda_{2}-\lambda_{3})}{\lambda_{1}+\lambda_{2}+\lambda_{3}},\;c_{s}=\frac{3\;\lambda_{3}}{\lambda_{1}+\lambda_{2}+\lambda_{3}},$$

where $\lambda_{i}$ is the *i*th eigenvalue of $D_{\mathrm{water}}\left(x\right)$, a≥1 is a multiplicative factor introduced to increase the anisotropy of the tensor, and $c_{l}$, $c_{p}$, and $c_{s}$ are the linear, planar, and spherical anisotropy measures of $D_{\mathrm{water}}\left(x\right)$, respectively.

**Figure 2:**
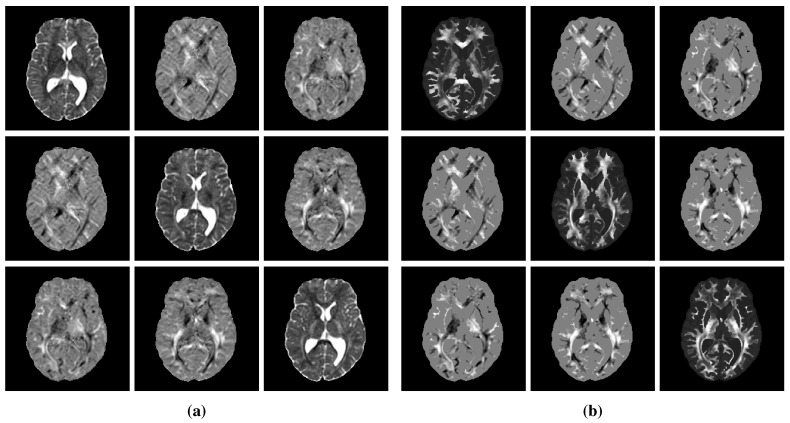
Example of processed DTI data. (**a**) DTI-derived water diffusion tensor field after susceptibility-induced distortion, eddy currents, and patient motion correction using FSL [33]. (**b**) Tumor diffusion tensor field with increased anisotropy in white matter (a=10) and scaled diffusivity ($d_{\mathrm{gray}}/d_{\mathrm{white}}=0.1$) built from the water diffusion tensor field in panel (**a**) and the brain map in Figure 1c as described in Section 2.3.6. The subpanel located at row *i* and column *j* of panels (**a**) and (**b**) corresponds to the tensor component $d_{i,j}$.

For reasons that will become clearer in Section 2.4, a unit tumor cell diffusion tensor was built at this stage by fixing $d_{\mathrm{white}}$ to 1. A value of 0.1 was chosen for the $d_{\mathrm{gray}}/d_{\mathrm{white}}$ ratio, as proposed previously in [13] to account for the restricted migration of tumor cells in gray compared to white matter. This ratio was supposed constant among all subjects as it is expected to depend exclusively on the structural organization of healthy white versus gray matter and not on the tumor characteristics. Similarly, the anisotropy factor *a* was fixed to 10 for all subjects, as suggested in [14]. An example of processed DTI data is depicted in Figure 2. The processed MR data of the 6 volunteers used in this study are publicly available at https://doi.org/10.5281/zenodo.6563613 (accessed on 17 May 2022). Further details on these data are available in Appendix B.

### 2.4. Dataset Synthesis

A synthetic tumor dataset was generated from the processed MR data described hereabove. 200 fictitious tumors were grown over the segmented brain domain of each of the 6 volunteers from randomly picked seeds and parameter values using the reaction-diffusion model in Equations (4)–(6), totaling 1200 synthetic tumors. For each tumor, the cell-density distribution was sampled at four imaging time points $t_{1}$, $t_{2}$, $t_{3}$, and $t_{4}$.

Each tumor seed consisted of a 3voxel×3voxel×3voxel neighborhood chosen among all segmented white matter voxels whose initial (i.e., at time $t=t_{0}$) normalized tumor cell-density *c* was set to 1. For each simulated tumor, an infiltration depth $\lambda =\sqrt{d_{\mathrm{white}}/\rho }$, a tumor front propagation speed $v=2\sqrt{d_{\mathrm{white}}\;\rho }$, and two imaging time intervals $\Delta t_{1}$ and $\Delta t_{2}$ were randomly chosen from uniform distributions (floating point for λ and *v*, integer for $\Delta t_{1}$ and $\Delta t_{2}$). The value ranges of the uniform distributions are provided in Table 1 and are of the same order of magnitude as in [19].

Two additional time intervals, $\Delta t_{3}$ and $\Delta t_{4}$, were fixed to 90 d for verification purposes (see Section 2.6). Starting from tumor emergence time $t_{0}$, the time intervals $\Delta t_{1-4}$ univocally define the four imaging time points $t_{i}=t_{0}+\sum_{j=1}^{i}\Delta t_{j},i=1,\ldots ,4$.

For each sampled couple of (λ,v) values, a white matter diffusion rate value $d_{\mathrm{white}}$ and a proliferation rate value ρ were derived as:

(12) $$\begin{array}{cc} d_{\mathrm{white}} & =\frac{\lambda \;v}{2} \end{array}$$

(13) $$\begin{array}{cc} \rho & =\frac{v}{2\;\lambda } \end{array}$$

**Figure 3:**
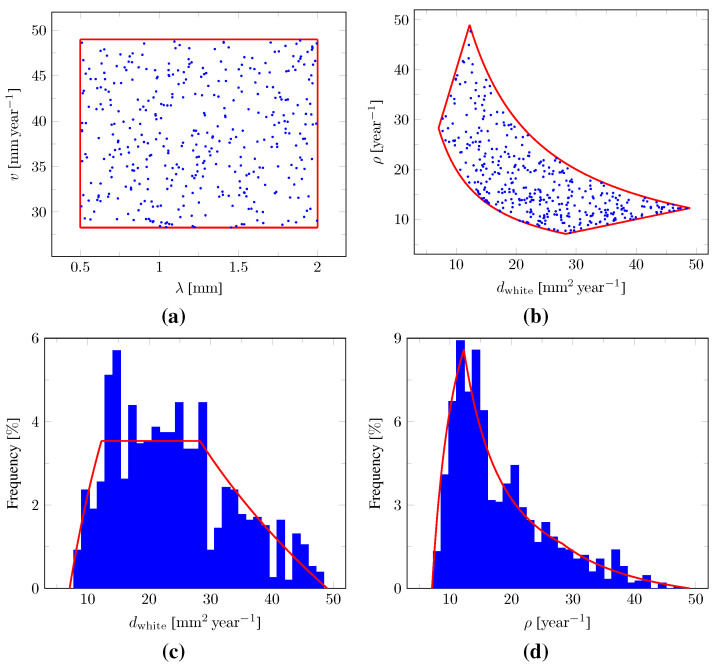
Sampling of the model parameters. (**a**) Empirical joint distribution of the (λ,v) values sampled from uniform distributions (blue marks) with superimposed sampling domain boundary (red segments). (**b**) Corresponding joint distribution of the derived $\left(d_{\mathrm{white}},\rho \right)$ values using Equations (12) and (13) (blue marks) with superimposed sampling domain boundary (red curves). (**c**) Empirical marginal distribution of the derived $d_{\mathrm{white}}$ values (blue bars) with superimposed theoretical distribution (red curves). (**d**) Empirical marginal distribution of the derived ρ values (blue bars) with superimposed theoretical distribution (red curves).

In this manner, a wide diversity of tumors can be uniformly sampled within a realistic range of infiltration depths λ and propagation speeds *v*. Independently sampling $d_{\mathrm{white}}$ and ρ values from uniform distributions may instead have resulted in tumors that are too small (i.e., with too small $d_{\mathrm{white}}$ and ρ values resulting in an empty $\Gamma_{1}$ contour, see Section 2.5.1 and Section 2.5.2) or too large (i.e., with too large $d_{\mathrm{white}}$ and ρ values resulting in a brain domain almost filled with tumor cells). The empirical joint distribution of the sampled (λ,v) values as well as the corresponding joint distribution of $\left(d_{\mathrm{white}},\rho \right)$ and marginal distributions of $d_{\mathrm{white}}$ and ρ are depicted in Figure 3.

**Figure 4:**
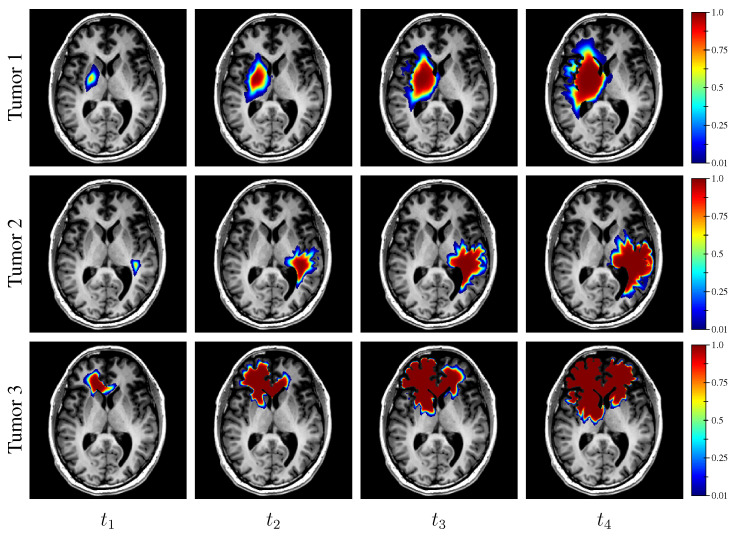
Examples of simulated tumor cell-density distributions at times $t_{1-4}$ (1st to 4th columns, axial slices) from the MR data of the same subject as in Figure 1 and Figure 2. The corresponding model parameter values are provided in Table 2.

For each synthetic tumor, a tumor cell diffusion tensor field D(x) was then obtained by multiplying the unit (unscaled) diffusion tensor derived as described in Section 2.3.6 by the derived value of $d_{\mathrm{white}}$. As a reminder, the ratio $d_{\mathrm{gray}}/d_{\mathrm{white}}$ was considered constant among tumors in this work (see Section 2.3.6). A tumor was finally grown from the sampled seed, tumor cell diffusion tensor field D(x), and proliferation rate ρ using the model and the simulated tumor cell distributions at times $t_{1-4}$ were stored. Examples of synthetic tumors are depicted in Figure 4. The corresponding model parameter values are provided in Table 2.

The model was solved by a forward Euler finite difference approach using a GPU implementation of the 3D standard stencil referenced in [41] for the computation of the divergence term in Equation (4). A time step Δt=0.5 d was chosen, ensuring the numerical stability of the method within the sampled parameter range. The processing time was around 1 ms per iteration on a GeForce GTX 1080 GPU (NVIDIA, Santa Clara, CA, USA), leading to total simulation times in range 0.72–1.08 s per tumor. All simulations were performed using a Python wrapping of our Tumour Growth Simulation ToolKit (TGSTK) written in C++/CUDA language based on VTK [36] and publicly available at https://github.com/cormarte/tgstk (accessed on 17 May 2022). The toolkit documentation can be found at https://cormarte.github.io/tgstk/html (accessed on 17 May 2022).

### 2.5. Deep Convolutional Neural Networks

Two deep convolutional neural networks were implemented and trained to respectively address the cell-density estimation and model parameter estimation problems introduced hereabove. All training steps were performed using the TensorFlow framework (version 2.5.0) [42] in Python on a GeForce RTX 3090 GPU (NVIDIA, USA). The Python code used for dataset generation and network training is available at https://github.com/cormarte/DeepLearningGliomaGrowthModeling (accessed on 17 May 2022).

#### 2.5.1. Cell-Density Estimation

The first problem addressed was to reconstruct a tumor cell-density distribution from (i) two imaging contours—$\Gamma_{1}$ and $\Gamma_{2}$—obtained through an imaging process described by Equation (2) for two detectability threshold values $c_{1}$ and $c_{2}$ at a given imaging time and (ii) a unit (unscaled) tumor cell diffusion tensor field derived from DTI data as presented in Section 2.3.6.

This approach is motivated by the asymptotic properties of the traveling wave solution admitted by Equation (1) for constant coefficients and on the infinite cylinder, whose profile decreases exponentially with decay constant λ (see Section 1). For such a solution, the value of λ can be trivially estimated given the distance between two cell-density iso-contours, and an approximate tumor cell-density distribution can subsequently be reconstructed for a sufficiently large distance to the tumor core. Here, we assess the ability of deep neural networks to build an approximate solution of Equations (4)–(6) in the more general case of a complex domain and variable anisotropic diffusion tensor field, and from 2 imaging contours obtained by Equation (2)—which do not necessarily coincide with cell-density iso-contours as discussed previously [20].

In a clinical setting, the value of the white matter diffusion rate $d_{\mathrm{white}}$ used to scale the tumor cell diffusion tensor in Equation (9) is unknown, and its estimation will be addressed in the next section. Therefore this information is not considered for this problem. In contrast, the preferred migration directions of tumor cells along the white matter tracts can be assessed from clinical DTI acquisitions as described in Section 2.3.6 and may be used for the estimation of the tumor cell-density distribution. This motivates the introduction of the unit unscaled diffusion tensor field as an input of this problem, in addition to the $\Gamma_{1}$ and $\Gamma_{2}$ contours.

**Figure 5:**
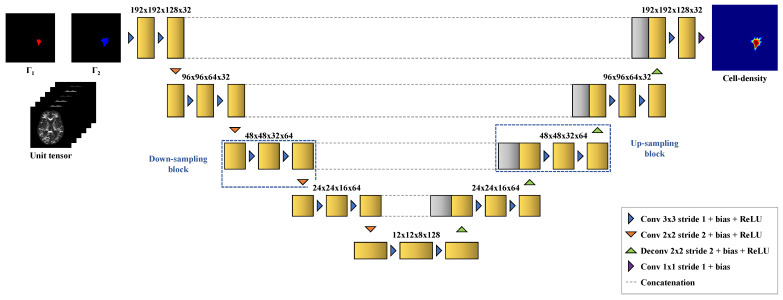
Three-dimensional U-Net architecture [43] with its feature map dimensions used for cell-density estimation. The network takes as input volumes of dimensions 192×192×128 with 8 channels corresponding to the 2 contours $\Gamma_{1}$ and $\Gamma_{2}$ and the 6 independent components of the unit (unscaled) tumor cell diffusion tensor field, and outputs a cell-density map with the same spatial dimensions.

To address this problem, a 3D DCNN based on the U-Net architecture [43] was implemented, as it has been successfully applied to many medical imaging problems previously [30,44]. The network consists of 4 down-sampling blocks, 4 up-sampling blocks, and 1 output block. Each down-sampling block is made of 2 convolutional layers with kernel size 3×3 and stride 1, followed by a bias-adding layer and a rectified linear unit (ReLU) activation layer. A convolutional layer with kernel size 2×2 and stride 2 is added at the end of the block to reduce the feature map dimensions by a factor 2. The up-sampling blocks are identical to the down-sampling blocks except that the last convolutional layer is replaced by a transposed convolution layer with kernel size 2×2 and stride 2, followed by a bias-adding layer and a ReLU activation layer to expand the feature map dimensions by a factor 2. Skip connections are added between the output of the second ReLU activation layer of each down-sampling block and the input of the corresponding upsampling block with the same spatial dimensions, implemented as a concatenation operation. The output block has the same structure as the down-sampling blocks except that the last convolutional layer is replaced by a convolutional layer with kernel size 1×1 and stride 1 followed by a bias-adding layer but no activation layer to merge the last 32 feature maps into a single tumor cell-density map. The network architecture with its feature map dimensions is depicted in Figure 5.

The network takes as input tensors of shape 192×192×128×8 (width × height × depth × channels). The first 2 channels are fed with the two binary regions respectively delimited by $\Gamma_{1}$ and $\Gamma_{2}$. These regions were obtained by thresholding each generated tumor cell distribution at the second imaging time point $t_{2}$ with threshold values $c_{1}$ and $c_{2}$ of 0.80 and 0.16, as previously proposed in [8] for the enhancing core and edema outlines, respectively. The last 6 channels correspond to the 6 independent components of the unit (unscaled) tumor cell diffusion tensor (see Section 2.3.6).

To evaluate the generalization ability of the model, the dataset was further split into training and test sets in proportion 83–17% on a patient basis (i.e., the 200 tumors generated from the MR data of the last patient are kept for evaluation purposes). The network was trained using the Adam optimizer [45] (learning rate: 10^−4^, $\beta_{1}$: 0.9, $\beta_{2}$: 0.999, ϵ: 10^−6^) and the mean absolute error (MAE) loss over mini-batches of size 1. Data augmentation was performed by applying random shifts in range ±15voxel in the three spatial dimensions to each batch. Rotations were not applied as they would also imply transformation of the tensor components [46], resulting in longer execution times for on-the-fly augmentation or larger dataset size for offline augmentation. The training was stopped early after no improvement in the test loss for 100 epochs. The network parameter values that provided the best test loss value (MAE = 1.24 × 10 ^−4^) were kept, which occurred after 876 epochs.

#### 2.5.2. Parameter Estimation

The second problem addressed is to estimate the value of the model parameters $d_{\mathrm{white}}$ and ρ (or equivalently of the derived parameters $\lambda =\sqrt{d_{\mathrm{white}}/\rho }$, and $v=2\sqrt{d_{\mathrm{white}}\;\rho }$) from (i) three imaging contours: two imaging contours—$\Gamma_{1}$ and $\Gamma_{2}$—obtained by Equation (2) for two different threshold values $c_{1}$ and $c_{2}$ at imaging time $t_{2}$ and a third imaging contour $\Gamma_{3}$ obtained for the same $c_{2}$ threshold value at the earlier imaging time $t_{1}$ and (ii) the unit (unscaled) tumor cell diffusion tensor field. The time interval $\Delta t_{2}$ between $t_{1}$ and $t_{2}$ is also considered as an input of the problem.

As for the cell-density estimation problem, the motivation for such inputs lies in the properties of the asymptotic traveling wave solution of Equation (1) (see Section 1), whose profile decay constant $\lambda =\sqrt{d/\rho }$ can be assessed from two cell-density iso-contours at a given time point as mentioned hereabove. In addition, the propagation speed of the tumor front $v=2\sqrt{d\;\rho }$ can similarly be assessed from the distance between a same cell-density iso-contour taken at two different time points given their temporal spacing. The knowledge of λ and *v* can finally be used to assess the individual values of *d* and ρ. Here again, we assess the ability of deep neural networks to generalize these properties in the case of a complex domain and variable anisotropic diffusion tensor field from 3 threshold-like imaging contours obtained by Equation (2). The same remark as for the previous section holds regarding the possibility of deriving a unit tumor diffusion tensor from DTI data, which can provide additional information for the estimation of the steepness and speed of the tumor front.

The DCNN implemented for this task is a convolutional encoder. The network consists of 6 convolutional down-sampling blocks and a fully connected output block. Each down-sampling block is made of 2 convolutional layers with kernel size 3×3 and stride 1, followed by a bias-adding layer and a ReLU activation layer. A convolutional layer with kernel size 2×2 and stride 2 is added at the end of the block to reduce the feature map dimensions by a factor 2. The output block flattens the 3×3×2×8 output of the last down-sampling block and concatenates a 1×1 (width × channels) tensor to the flattened vector to feed the imaging time interval $\Delta t_{2}$. A fully connected layer followed by a bias-adding layer but no activation layer is finally used to merge the last 145 components into 2 scalar values for λ and *v*.

The network takes as input tensors of shape 192×192×128×9 (width × height × depth × channels). The first 3 channels are fed with the binary regions respectively delimited by $\Gamma_{1}$, $\Gamma_{2}$, and $\Gamma_{3}$. These regions were respectively obtained by thresholding each generated tumor cell distribution at time $t_{2}$ with threshold values of 0.80 ($\Gamma_{1}$) and 0.16 ($\Gamma_{2}$), and the distribution at time $t_{1}$ with a value threshold value of 0.16 ($\Gamma_{3}$) [8]. The last 6 channels correspond to the 6 independent components of the unit (unscaled) tumor cell diffusion tensor (see Section 2.3.6). To account for their different value range and scale, the target values of λ and *v* were standardized using the theoretical mean and variance of the respective uniform distributions from which they were sampled.

The same training/test splitting as for the tumor cell-density estimation network was applied to the dataset. The network was trained using the Adam optimizer [45] (learning rate: 10^−4^, $\beta_{1}$: 0.9, $\beta_{2}$: 0.999, ϵ: 10^−6^) and the mean squared error (MSE) loss. Data augmentation was performed by applying random shifts in range ±15voxel in the three spatial dimensions to each input batch. Early stopping was applied if no improvement was observed in the test loss for 100 epochs. The network parameter values that provided the best test loss value (MSE = 6.75 × 10 ^−2^) were kept, which occurred after 628 epochs.

### 2.6. Verification

**Figure 6:**
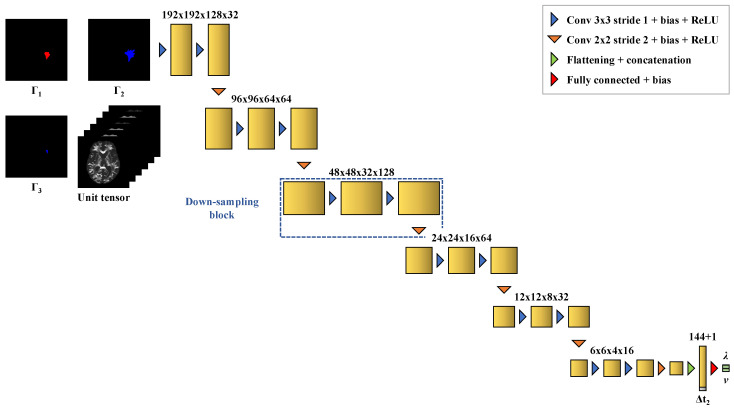
Three-dimensional convolutional regressor architecture with its feature map dimensions used for parameter estimation. The network takes as input volumes of dimensions 192×192×128 with 9 channels corresponding to the 3 contours $\Gamma_{1}$, $\Gamma_{2}$, and $\Gamma_{3}$ and the 6 independent components of the unit (unscaled) tumor cell diffusion tensor field as well as the time interval $\Delta t_{2}$ between $\Gamma_{3}$ and $\Gamma_{2}$, and outputs estimated values of λ and *v*.

To verify and illustrate our approach, we conducted the following numerical experiment: Starting from the tumor cell-density distribution estimated at time $t_{2}$ from $\Gamma_{1}$ and $\Gamma_{2}$ using our first network (Figure 5), as well as the values of $d_{\mathrm{white}}$ and ρ estimated from $\Gamma_{1}$, $\Gamma_{2}$, and $\Gamma_{3}$ using our second network (Figure 6) and Equations (12) and (13), we computed a tumor cell-density distribution using the reaction-diffusion model at times $t_{3}$ and $t_{4}$, 90 d and 180 d later, respectively. We then compared the estimated distributions to the actual tumor cell-density distributions at times $t_{3}$ and $t_{4}$—i.e., those obtained for the true cell-density distribution at time $t_{2}$ as well as the true values of $d_{\mathrm{white}}$ and ρ—using the MAE computed voxelwise within the c>0.01 contour. This latter restriction prevents background or weakly invaded voxels to artificially lower the MAE. The Hausdorff distance $d_{H}$ and the average symmetric surface distance (ASSD) $d_{S}$ between the imaging contours obtained from the true and estimated tumor cell-density distributions for threshold values of $c_{1}$ and $c_{2}$ were also computed for each test tumor and time point, as given by:

(14) $$\begin{array}{cc} \;d_{H}\left(A,B\right) & =\max\left\{\max_{b\in B}\left\{\min_{a\in A}d\left(a,b\right)\right\},\max_{a\in A}\left\{\min_{b\in B}d\left(a,b\right)\right\}\right\}, \end{array}$$

(15) $$\begin{array}{cc} d_{S}\left(A,B\right) & =\frac{1}{\left|A|+|B\right|}\left(\sum_{b\in B}\min_{a\in A}d\left(a,b\right)+\sum_{a\in A}\min_{b\in B}d\left(a,b\right)\right), \end{array}$$

where d(a,b) is the Euclidian distance between elements *a* and *b*, and |X| is the cardinal of set *X*.

It should be noted that minor post-processing was applied to the estimated tumor cell-density distributions at time $t_{2}$ provided by the first network prior to the computation of the densities at times $t_{3}$ and $t_{4}$. First, the cell-density of non-brain voxels (i.e., cerebrospinal fluid and background voxels) was set to 0. Indeed, small (∼10^−5^) but non-zero values were observed for some of these voxels in the predicted tumor cell-density distributions. Second, maximum densities were clipped to 1 as small overshootings were also occasionally observed. Third, voxels located outside the largest connected region with densities above 1 × 10^−6^ were also set to 0 since small local maxima (∼10^−5^) were sporadically observed far from the tumor core, which gave rise to new tumor foci throughout the simulation. These post-processing steps allow us to correct for inaccuracies in the non-constrained output of our convolutional network and ensure numerical stability of the reaction-diffusion model solution at later times.

Sensitivity analyses of our approach were also performed by evaluating both network outputs on the test set after application of a systematic variation of ±10% on the threshold values $c_{1}$ and $c_{2}$ used to generate the $\Gamma_{1}$, $\Gamma_{2}$, and $\Gamma_{3}$ input contours, reflecting the inherent uncertainties in these values.

Finally, to demonstrate the applicability of our approach in a clinical context, a cell-density map was generated from the retrospective MR data of the GBM patient (see Section 2.2 and Section 2.3). To this extent, the segmented enhancing core and edema regions (see Section 2.3.5) were provided to the first network along with the derived unit tumor cell diffusion tensor.

## 3. Results

**Figure 7:**
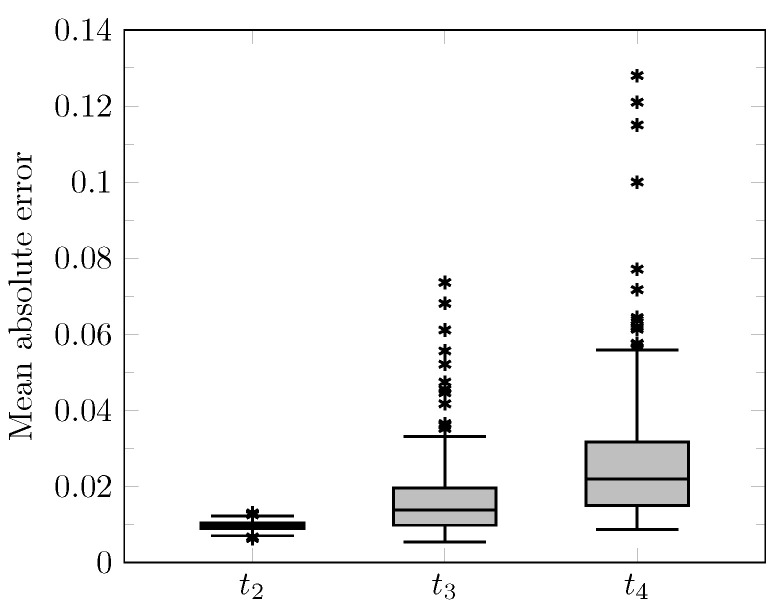
Boxplots of the mean absolute error distribution within the c>0.01 contour computed voxelwise over the whole test set for times $t_{2}=\Delta t_{1}+\Delta t_{2}\in [180,\;360]d$ (see Table 1), $t_{3}=t_{2}+90d$, and $t_{4}=t_{2}+180d$. Horizontal line: median, box: interquartile range, whiskers: ±1.5 interquartile range, asterisks: outliers.

**Figure 8:**
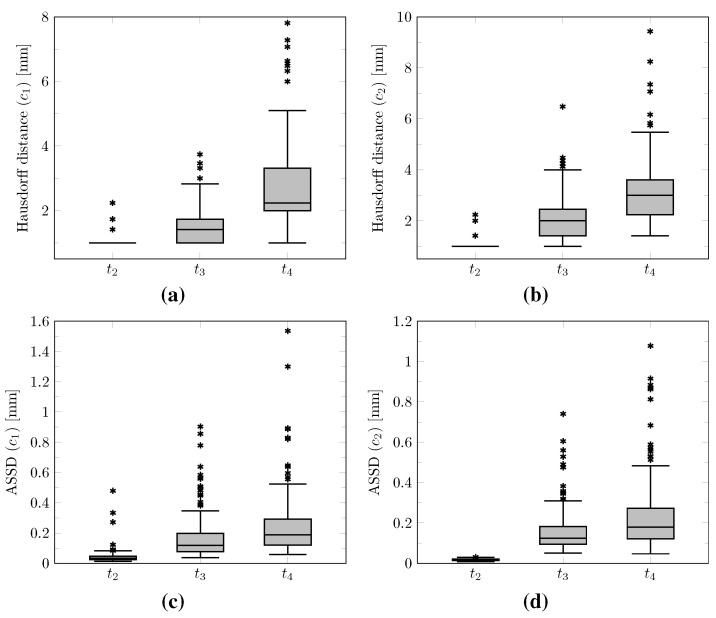
Boxplots of the Hausdorff distance and average symmetric surface distance (ASSD) distributions computed between the true and estimated imaging contours over the whole test set for times $t_{2}=\Delta t_{1}+\Delta t_{2}\in [180,\;360]\;d$ (see Table 1), $t_{3}=t_{2}+90\;d$, and $t_{4}=t_{2}+180\;d$. (**a**,**b**) Hausdorff distances computed between the true and estimated imaging contours obtained for threshold values of $c_{1}=0.80$ and $c_{2}=0.16$, respectively. (**c**,**d**) ASSD values computed between the true and estimated imaging contours obtained for threshold values of $c_{1}=0.80$ and $c_{2}=0.16$, respectively. Horizontal line: median, box: interquartile range, whiskers: ±1.5 interquartile range, asterisks: outliers.

**Figure 9:**
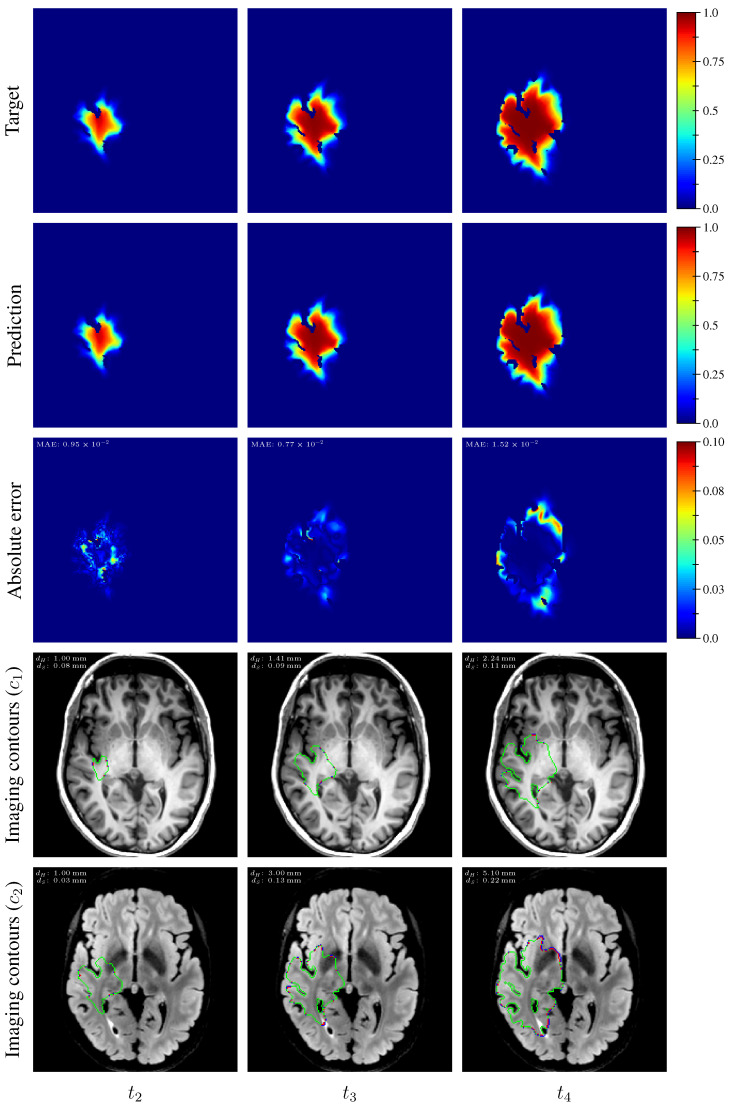
Example of true (1st row) and estimated (2nd row) three-dimensional tumor cell-density distributions at times $t_{2-4}$ (1st to 3rd column, axial slices) along with the corresponding absolute error maps (3rd row) for a test tumor ($d=43.47\;{\mathrm{mm}}^{2}{\mathrm{year}}^{-1}$, $\rho =11.22\;{\mathrm{year}}^{-1}$, $t_{1}=94\;d$, $t_{2}=264\;d$). The imaging contours for threshold values $c_{1}=0.80$ and $c_{2}=0.16$ superimposed to the T1 and T2 FLAIR image are depicted in the 4th and 5th rows, respectively. The blue, red, and green segments respectively correspond to the target, prediction, and overlapping contour voxels. MAE: mean absolute error for c>0.01, $d_{H}$: Hausdorff distance, $d_{S}$: average symmetric surface distance.

The distribution of the mean absolute error computed over the test set between the true and estimated tumor cell-density distributions at time $t_{2}$ within the c>0.01 contour is summarized by a boxplot in Figure 7 (first plot). Boxplots of the Hausdorff distance and ASSD distributions computed over the test set between the true and estimated imaging contours at time $t_{2}$ for threshold values $c_{1}=0.80$ and $c_{2}=0.16$ are provided in Figure 8 (first plots). The corresponding median values are provided in Table 3. An example of true and estimated tumor cell-density distributions at time $t_{2}$ from the test set is depicted in Figure 9 (first column), along with the corresponding absolute error map as well as the true and estimated imaging contours for threshold values $c_{1}=0.80$ and $c_{2}=0.16$. Additional examples are provided in Appendix C. All predicted tumor cell-density distributions at time $t_{2}$ used in Figure 7, Figure 8 and Figure 9 were provided by the first network (Figure 5).

**Figure 10:**
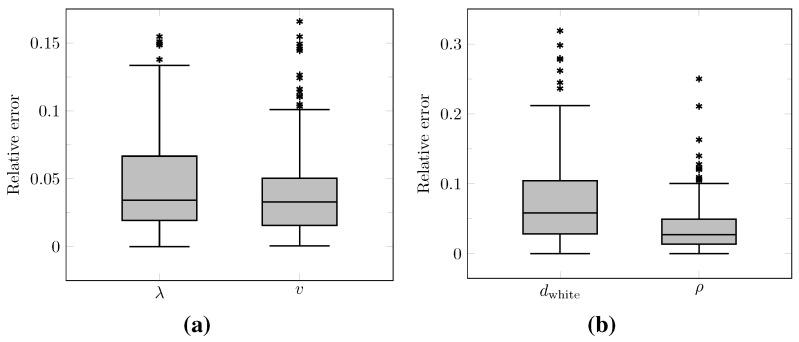
Boxplots of the relative error on the predicted model parameter values evaluated on the test set. (**a**) Relative errors on the estimated values of λ and *v* provided by the second network (Figure 6). (**b**) Corresponding relative errors on the derived values of $d_{\mathrm{white}}$ and ρ using Equations (12) and (13). Horizontal line: median, box: interquartile range, whiskers: ±1.5 interquartile range, asterisks: outliers.

**Figure 11:**
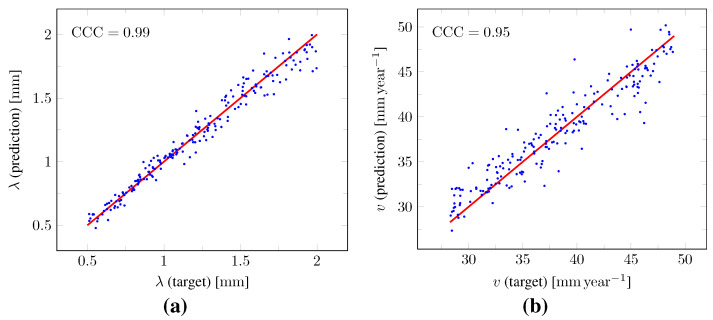

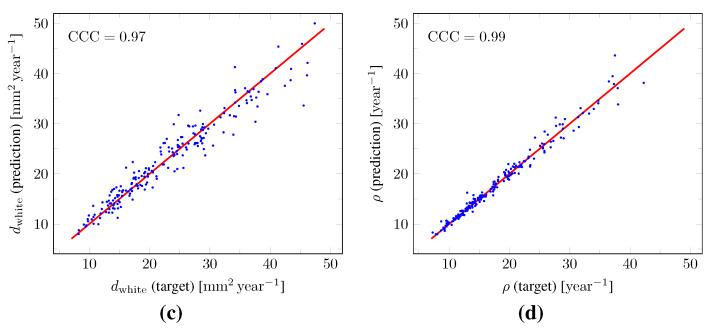
Scatterplots of the true versus predicted values of the model parameters from the test set. (**a**,**b**) True versus predicted values of λ and *v* provided by the second network (Figure 6). (**c**,**d**) True versus estimated values of $d_{\mathrm{white}}$ and ρ derived from the predicted values of λ and *v* using Equations (12) and (13). For each plot, the identity function is superimposed in red and the corresponding Lin’s concordance correlation coefficient (CCC) [47] is provided.

The distributions of the relative error on the values of λ and *v* computed at time $t_{2}$ over the test set as well as on the values of $d_{\mathrm{white}}$ and ρ derived with Equations (12) and (13) are summarized by boxplots in Figure 10. The corresponding median relative errors were 3.41%, 3.30%, 5.86%, and 2.75% for λ, *v*, $d_{\mathrm{white}}$, and ρ, respectively. The true versus predicted values of λ and *v* as well as of $d_{\mathrm{white}}$ and ρ from the test set are plotted in Figure 11. The corresponding Lin’s concordance correlation coefficients (CCC) [47] were 0.99, 0.95, 0.97, and 0.99 for λ, *v*, $d_{\mathrm{white}}$, and ρ, respectively.

As for imaging time $t_{2}$, the distributions of the mean absolute error computed over the test set between the true and estimated tumor cell distributions at times $t_{3}$ and $t_{4}$ within the c>0.01 contour are summarized by boxplots in Figure 7 (second and third plot, respectively). Boxplots of the Hausdorff distance and ASSD distributions computed over the test set between the true and estimated imaging contours at times $t_{3}$ and $t_{4}$ for threshold values $c_{1}=0.80$ and $c_{2}=0.16$ are also provided in Figure 8 (second and third plots). The corresponding median values are provided in Table 3. The true and estimated tumor cell-density distributions at times $t_{3}$ and $t_{4}$ are depicted in Figure 9 (second and third column, respectively) for the same test case as for time $t_{2}$, along with the corresponding absolute error maps as well as the true and estimated imaging contours for threshold values $c_{1}=0.80$ and $c_{2}=0.16$. Additional examples are provided in Appendix C. A loss of accuracy in the estimated tumor cell-density distributions over simulated time is observed in Figure 7, Figure 8 and Figure 9 and Table 3. The estimated tumor cell-density distributions at times $t_{3}$ and $t_{4}$ used in Figure 7, Figure 8 and Figure 9 and Table 3 were computed using the reaction-diffusion model as described in Section 2.6 from (i) the cell-density distribution predicted at time $t_{2}$ provided the first network (Figure 5) and (ii) the predicted model parameter values provided by the second network (Figure 6).

The results of the sensitivity analyses are summarized in Table 4 and Table 5. Performance indices are reported for all possible combinations of ±10% perturbations on the $c_{1}$ and $c_{2}$ values used to generate the input $\Gamma_{1}$, $\Gamma_{2}$, and $\Gamma_{3}$ imaging contours of both CNNs.

**Figure 12:**
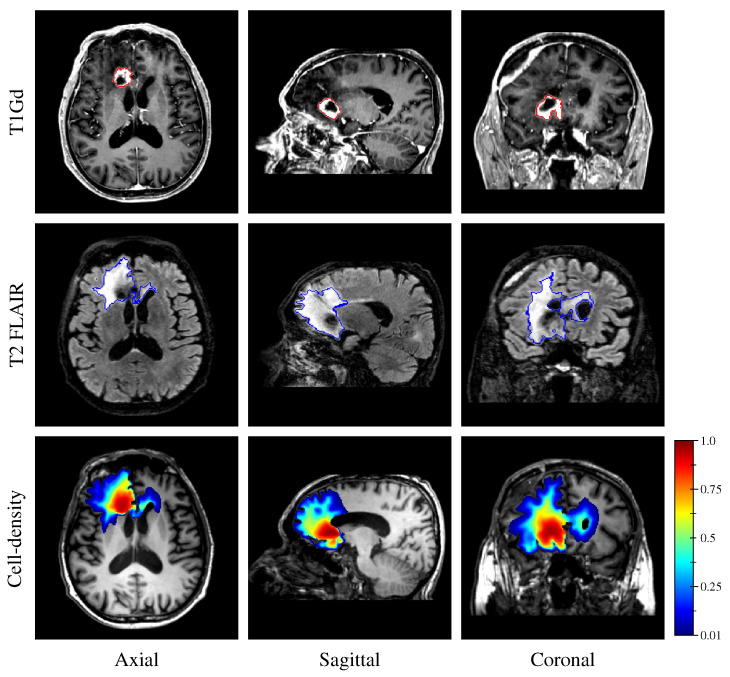
T1Gd image (1st row), T2 FLAIR image (2nd row), and estimated three-dimensional tumor cell-density distribution using the first network (3rd row) for an IDH-wildtype glioblastoma patient in axial (1st column), sagittal (2nd column), and coronal (3rd column) planes. The contours of the segmented enhancing core and peritumor vasogenic edema are superimposed in red on the T1Gd (1st row) and in blue on the T2 FLAIR (2nd row) images, respectively.

Finally, the estimated tumor cell-density distribution for the studied GBM patient provided by the first network (see Figure 5) is depicted in Figure 12 along with the T1Gd and T2 FLAIR images with superimposed segmented enhancing core and edema contours, respectively.

## 4. Discussion

Reaction-diffusion models have been studied for decades to capture the growth of gliomas, but the ill-posedness of their initialization at imaging time and estimation of their parameter values has restrained their use as a personalized predictive clinical tool. In this work, we showed the ability of DCNNs to circumvent these limitations, opening a wide range of opportunities in the field. Our approach only requires (i) deriving a unit diffusion tensor field from clinical DTI data as described herein, accounting for the preferential migration of tumor cells along white matter tracts and (ii) extracting three imaging contours obtained through a cell-density threshold-like process described by Equation (2) for two different threshold values and time points.

Regarding the second requirement, the outlines of the peritumor vasogenic edema and enhancing core have been proposed previously [8], visible on T2 FLAIR and T1Gd MR images acquired in routine for glioma follow-up, respectively. Nevertheless, it is worth noticing that peritumor vasogenic edema does not strictly speaking correspond to a region of tumor cell invasion but results from an accumulation of extracellular fluid originating from tumor-induced alterations of the blood–brain barrier [48,49] and changes in hydrodynamic pressure [50]. Consequently, the T2 FLAIR imaging process might not be accurately described by Equation (2), as also supported by our previous histological analysis in [20]. Furthermore, anti-angiogenic drugs are known to dramatically reduce vasogenic edema without however stopping tumor progression [48]. Therefore, other MR sequences or modalities could be better suited for the estimation of the tumor cell-density distribution and parameters of reaction-diffusion glioma growth models. For instance, ADC maps derived from DW-MRI data could more accurately reflect tumor cell invasion, as proposed in [18,51]. PET imaging with radio-labeled amino acids could also provide additional information to this extent, as suggested in [26,52].

Once the aforementioned prerequisites are met, our approach makes it possible to (i) extrapolate a whole brain-tumor cell-density distribution within and beyond the visible outlines of the tumor that is compatible with the reaction-diffusion model in Equations (4)–(6) and (ii) individually assess the value of the diffusion and proliferation parameters of the model. Extrapolating tumor invasion is of utmost interest for radiotherapy planning since it would allow us to define personalized margins which more accurately target the tumor while avoiding irradiation of the healthy tissues, as previously discussed in [2,10]. The independent assessment of the diffusivity and proliferation parameters of the model is for its part of great interest to better characterize the tumor [22]. The combination of both gives access to a fully personalized tool, initialized from clinical imaging data and allowing us to anticipate the spatial–temporal growth of gliomas. Such a tool could, for example, be of considerable interest for dose fractionation optimization in radiotherapy using a reinforcement learning approach, as used in [53]. Furthermore, as it only depends on post-processed data (binary segmentations and a DTI-derived water diffusion tensor) rather than raw MR data, the proposed approach may be robustly extended to other scanners and centers. In addition, the method is by design robust to variations in the time interval between the two required MR acquisitions since the interval is provided as an input of the second network for the estimation of the model parameters, which makes it well-adapted to the clinical reality.

The proposed method was found to provide accurate estimations of the three-dimensional tumor cell distribution from only two imaging contours at a single time point, with a median voxelwise MAE below 10^−2^ within the c>0.01 contour—as evaluated on 200 synthetic tumors grown over the real brain domain of a test subject not used for network training. Our method also provided accurate estimates of the individual diffusion and proliferation parameters of the model from three imaging contours extracted from two time points for the same test tumors, with median relative errors of 5.86% and 2.75%, respectively (see Figure 10), and strong concordance (CCC≥0.95) with the true parameter values (see Figure 11). Furthermore, we showed that the spatio-temporal evolution of the tumor cell-density distribution at later time points (90 d and 180 d later) can be accurately captured from the estimated distribution at imaging time and parameter values using the reaction-diffusion model. The ASSD between the true and estimated imaging contours obtained for threshold values of $c_{1}=0.80$ and $c_{2}=0.16$ were indeed found to be lower than or equal to the pixel spacing (1mm×1mm×1mm) in most cases (see Figure 8). Nevertheless, a loss of accuracy in the estimated tumor cell-density over simulated time was observed (see Figure 7, Figure 8 and Figure 9 and Table 3), imputed to the amplification of errors originating from uncertainties in the estimated model parameter values and tumor cell-density distribution at imaging time. In particular, artefactual local maxima in the tumor cell-density distributions predicted by the CNN were found to give rise to new tumor foci over time. Post-processing steps were introduced to circumvent these effects (see Section 2.6), but residual artefacts were still observed, resulting in a large Hausdorff distance though small ASSD values for a few isolated cases (see outliers in Figure 8a,b). Our approach was also found to be robust to uncertainties in the tumor cell-density threshold values defining the input imaging contours of both CNNs. Indeed, all combinations of ±10% perturbations on both threshold values used to generate the contours resulted in an increase in median relative error within reasonable ranges of 14.82–22.39% and 10.25–17.79% for the diffusivity and proliferation rate, respectively. Finally, we also demonstrated the applicability of our proposed method to actual MR data of a GBM patient, for which we were able to reconstruct a tumor cell-density distribution compatible with the imaging data. Nevertheless, the lack of biopsy samples combined with the multiple treatments undergone by the patient prevented the validation of the estimated distribution, which was left for a future prospective study.

Compared to the current state-of-the-art approach in [27], our method appears to perform at least as well or better in most cases on the model parameter estimation problem, by comparison of the reported relative errors on the parameter values in [27]. Nevertheless, both methods were not evaluated on the same synthetic tumor dataset and no open-source code was available in [27] for further comparison. Besides, once trained, the CNNs used in this work no longer depend on any arbitrary parameters, as opposed to the method in [27] for which adequate sparsity level and observation operator weighting need to be selected, in addition to the many parameters involved in the numerical scheme (Gaussian standard deviation, coarsening levels, initial solution guesses, tolerance thresholds, termination criteria, …) [27,29]. Our method also performs significantly faster: parameter estimation on a single case is performed within around 0.1 s versus 1000 s for [27], which however remains largely sufficient for the application in both cases. Furthermore, the proposed approach allows for absolute parameter estimation, as opposed to [27] which only provides non-dimensionalized estimates that could not be scaled as the time between tumor emergence and imaging is actually not known. Consequently, comparison of the estimated parameter values between tumors or their application to predict tumor evolution over time using the model are prevented. Nevertheless, contrary to [27], our method requires two imaging time points to compensate for the ill-posedness of the problem and to allow for dimensionalized parameter estimation—but in return makes no explicit assumption on the initial tumor cell distribution. This latter requirement of our approach implies that the tumor diffusivity and proliferation rate remain constant between the scans—which is in any case also implicitly assumed between the tumor emergence and the single scan time for the forward problem described in [27]. As a consequence, our method is expected to be sensitive to any treatment administered between the scans that would significantly impact the tumor model parameters (chemotherapy, radiotherapy) or solving domain (surgery). Finally, as opposed to [27], our method considers a spatially variable tumor cell diffusion tensor accounting for the preferential migration of tumor cells along white matter tracts, but is restricted to monofocal tumors in the present form.

As a future work, tumor-induced mass effect should be further integrated into the reaction-diffusion model since it is known to cause substantial deformations of the brain parenchyma and distortions of the white matter tracts as the tumor grows, which should also be taken into account for accurate treatment planning. Such effects have been previously considered [6,7], but would introduce additional parameters to be estimated. In addition, transient brain deformations would hardly be integrated into a regular grid-based approach such as the finite difference method used in this work without loss of precision. A finite element formulation over an unstructured mesh could be used instead but would be much more computationally expensive—hence less suited for the generation of large high-resolution datasets such as the one described herein. Tumor-induced destruction of the white matter tracts should also be further considered, as an accurate capture of the original orientation of the brain fibers within the tumor region is required for the evaluation of the cell-density distribution and model parameter values using both DCNNs. A solution to this problem has been previously proposed in [25]—though subject to limitations—in which symmetry of the brain is exploited to artificially reconstruct the missing brain fibers. More advanced methods should be investigated in this sense. Necrosis could also be integrated into the model as proposed in [15,16], which would have avoided the counter-intuitive correspondence between the hyper-dense (c∼1) region of the estimated tumor cell-density distribution and the necrotic area visible on MRI in Figure 12. Furthermore, the deep neural networks presented herein remain little flexible as they would need to be retrained if different imaging threshold values were considered, although transfer learning could be used to benefit from the lower-level features learned herein and avoid retraining the networks from scratch [54]. Ultimately, the threshold values could be fed to the networks along with the binary contours, but this would make the problem even more complex and would therefore require an even larger training dataset. Although real medical imaging data were used in this work, the verification of our approach still relied on healthy subject data. Therefore, the underlying hypothesis was made that the reaction-diffusion model defined by Equation (4)–(6) and used for tumor synthesis is indeed able to accurately capture the growth of real gliomas, which has never been extensively demonstrated so far to the best of our knowledge. Validation of our approach on actual glioma patient data should be further performed, but longitudinal imaging data with stereotactic biopsies of untreated glioma patients remain scarce. Including the effects of treatments into reaction-diffusion models has also been proposed previously [4,5,17,18,19], but again introduces additional parameters, increasing the complexity of the problem. Alternately, the method could be applied to large publicly available datasets such as the BRaTS dataset [28]. Whereas ground-truth cell-density distributions and model parameter values are unknown for such datasets, indirect validation by investigation of the predictive performance of the estimated model parameters for tumor bio-markers could still be performed, as attempted in [29].

This work also highlights the added value of DCNNs for the resolution of ill-posed problems that are hardly solved by classical optimization methods, and provides encouraging results towards the full personalization of reaction-diffusion glioma growth models from medical imaging data, which has remained unsolved for decades.

## 5. Conclusions

We proposed a deep learning-based approach to simultaneously address the problems of estimating the tumor cell-density distribution at diagnosis and parameter values of a reaction-diffusion glioma growth model from patient magnetic resonance imaging data. We demonstrated the accuracy of our approach on synthetic tumors grown over actual brain domains of healthy volunteers. We also showed the applicability of our method on MR data of a real glioblastoma patient. Our promising results which point towards the full personalization of glioma reaction-diffusion models may open up tremendous possibilities in the field.

## Figures and Tables

**Table 1:** Value ranges and units of the uniform distributions used to sample the tumor growth model parameters for the generation of the synthetic tumor dataset.

	Min	Max	Units
λ	0.5	2.0	mm
v	28.28	48.99	$mm{\mathrm{year}}^{-1}$
$\Delta t_{1}$	90	180	d
$\Delta t_{2}$	90	180	d

**Table 2:** Parameter values used for the tumor simulations in Figure 4.

	$d_{\mathrm{white}}$ [${\mathrm{mm}}^{2}{\mathrm{year}}^{-1}$]	ρ [${\mathrm{year}}^{-1}$]	λ [mm]	*v* [$\mathrm{mm}{\mathrm{year}}^{-1}$]	$t_{1}$ [d]	$t_{2}$ [d]	$t_{3}$ [d]	$t_{4}$ [d]
**Tumor 1**	10.87	31.77	1.71	37.16	175	328	418	508
**Tumor 2**	15.07	13.95	0.96	29.00	146	316	406	496
**Tumor 3**	41.49	11.31	0.52	43.33	137	242	332	422

**Table 3:** Median values of the voxelwise mean absolute error (MAE) between the true and estimated cell-density distributions within the c>0.01 contour as well as of the Hausdorff distance and average symmetric surface distance (ASSD) between the true and estimated imaging contours for threshold values of $c_{1}=0.80$ and $c_{2}=0.16$ computed over the test set for times $t_{2}$, $t_{3}$, and $t_{4}$.

	$t_{2}$	$t_{3}$	$t_{4}$
**Median MAE [10^−2^]**	0.96	1.38	2.20
**Median Hausdorff ($c_{1}$) [mm]**	1.00	1.41	2.24
**Median Hausdorff ($c_{2}$) [mm]**	1.00	2.00	3.00
**Median ASSD ($c_{1}$) [mm]**	0.03	0.12	0.19
**Median ASSD ($c_{2}$) [mm]**	0.02	0.12	0.18

**Table 4:** Median relative errors (MRE) and Lin’s concordance correlation coefficients (CCC) [47] between the true and predicted values of λ and *v* provided by the second network (Figure 6) and of $d_{\mathrm{white}}$ and ρ derived using Equations (12) and (13), computed over the test set for all combinations of ±10% perturbations on the $c_{1}$ and $c_{2}$ threshold values used to generate the input contours $\Gamma_{1}$, $\Gamma_{2}$, and $\Gamma_{3}$.

	Perturbation on $c_{1}\;|\;c_{2}$							
	−10% |−10%		−10% |+10%		+10% |−10%		+10% |+10%	
	**MRE**	**CCC**	**MRE**	**CCC**	**MRE**	**CCC**	**MRE**	**CCC**
λ	12.51%	0.92	18.55%	0.83	25.54%	0.82	18.90%	0.88
v	3.48%	0.94	3.88%	0.93	4.62%	0.91	4.12%	0.93
$d_{\mathrm{white}}$	13.43%	0.90	19.93%	0.81	28.25%	0.80	22.47%	0.86
ρ	13.00%	0.94	20.54%	0.89	17.04%	0.86	13.31%	0.91

**Table 5:** Median values of the voxelwise mean absolute error (MAE) between the true and estimated cell-density distributions within the c>0.01 contour as well as of the Hausdorff distance and average symmetric surface distance (ASSD) between the true and estimated imaging contours for threshold values of $c_{1}=0.80$ and $c_{2}=0.16$ computed over the test set for times $t_{2}$, $t_{3}$, and $t_{4}$. The predicted cell-density estimations and parameter values used for the calculations were obtained for all combinations of ±10% perturbations on the $c_{1}$ and $c_{2}$ threshold values used to generate the input contours $\Gamma_{1}$, $\Gamma_{2}$, and $\Gamma_{3}$ of both networks.

	Perturbation on $c_{1}\;|\;c_{2}$											
	−10% |−10%			−10% |+10%			+10% |−10%			+10% |+10%		
	$t_{2}$	$t_{3}$	$t_{4}$	$t_{2}$	$t_{3}$	$t_{4}$	$t_{2}$	$t_{3}$	$t_{4}$	$t_{2}$	$t_{3}$	$t_{4}$
**Median MAE [10^−2^]**	3.10	3.14	3.38	2.94	2.95	3.26	3.70	3.41	3.63	3.86	3.46	3.57
**Median Hausdorff ($c_{1}$) [mm]**	2.24	2.45	3.32	2.24	2.45	3.32	3.0	3.0	3.32	3.0	3.0	3.67
**Median Hausdorff ($c_{2}$) [mm]**	1.00	2.00	3.00	1.0	2.0	3.0	1.0	2.0	3.16	1.0	2.0	3.16
**Median ASSD ($c_{1}$) [mm]**	0.63	0.51	0.39	0.63	0.49	0.39	0.90	0.56	0.40	0.90	0.61	0.43
**Median ASSD ($c_{2}$) [mm]**	0.12	0.16	0.19	0.11	0.14	0.18	0.12	0.17	0.24	0.11	0.15	0.22

**Figure A1:**
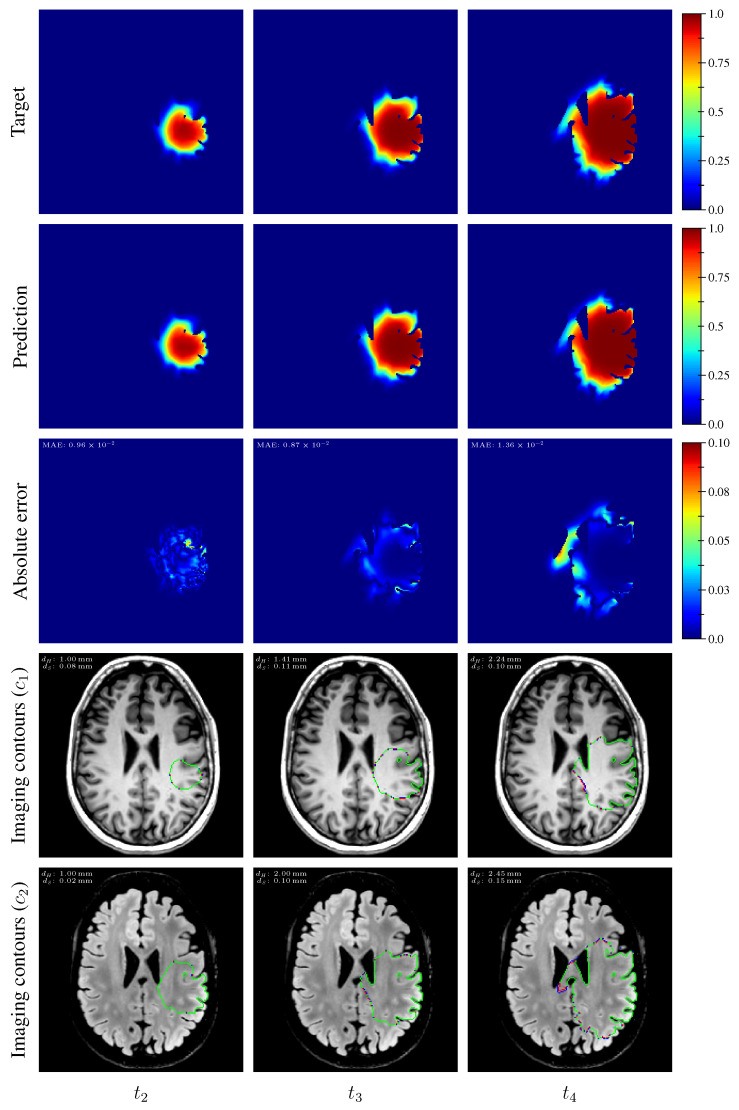
Example of true (1st row) and estimated (2nd row) three-dimensional tumor cell-density distributions at times $t_{2-4}$ (1st to 3rd column, axial slices) along with the corresponding absolute error maps (3rd row) for a test tumor ($d=46.20\;{\mathrm{mm}}^{2}{\mathrm{year}}^{-1}$, $\rho =12.92\;{\mathrm{year}}^{-1}$, $t_{1}=130\;d$, $t_{2}=268\;d$). The imaging contours for threshold values $c_{1}=0.80$ and $c_{2}=0.16$ superimposed to the T1 and T2 FLAIR image are depicted in the 4th and 5th rows, respectively. The blue, red, and green segments respectively correspond to the target, prediction, and overlapping contour voxels. MAE: mean absolute error for c>0.01, $d_{H}$: Hausdorff distance, $d_{S}$: average symmetric surface distance.

**Figure A2:**
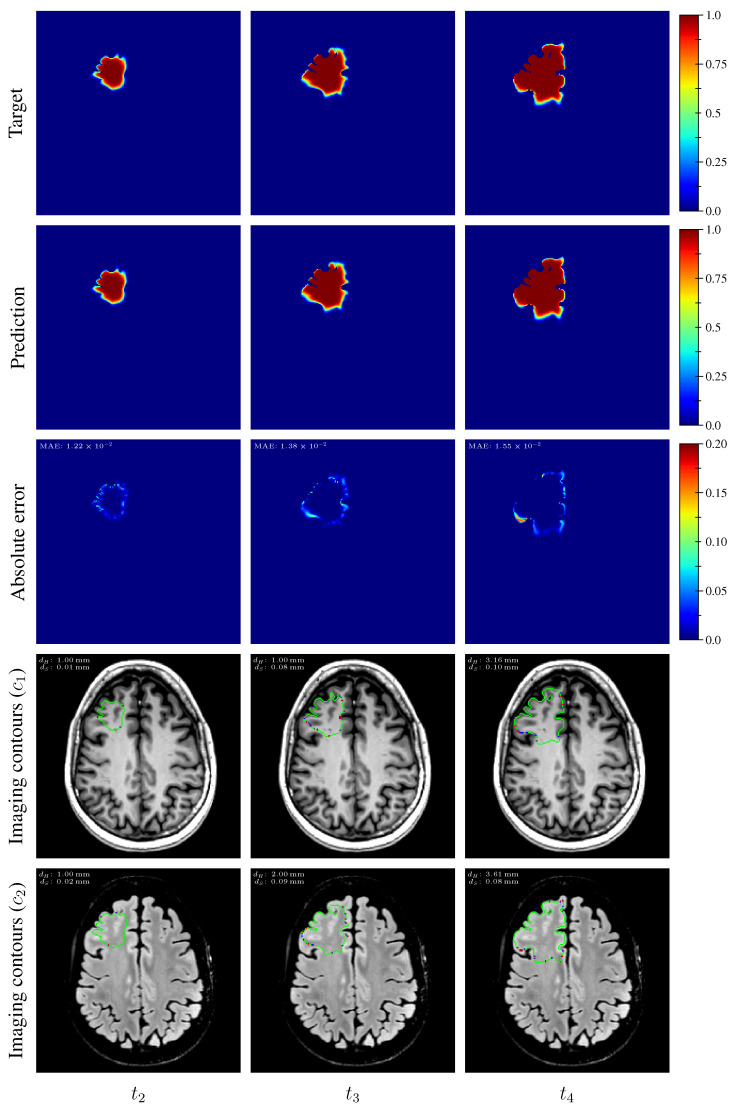
Example of true (1st row) and estimated (2nd row) three-dimensional tumor cell-density distributions at times $t_{2-4}$ (1st to 3rd column, axial slices) along with the corresponding absolute error maps (3rd row) for a test tumor ($d=9.52\;{\mathrm{mm}}^{2}{\mathrm{year}}^{-1}$, $\rho =25.77\;{\mathrm{year}}^{-1}$, $t_{1}=94\;d$, $t_{2}=225\;d$). The imaging contours for threshold values $c_{1}=0.80$ and $c_{2}=0.16$ superimposed to the T1 and T2 FLAIR image are depicted in the 4th and 5th rows, respectively. The blue, red, and green segments correspond to the target, prediction, and overlapping contour voxels, respectively. MAE: mean absolute error for c>0.01, $d_{H}$: Hausdorff distance, $d_{S}$: average symmetric surface distance.

## Data Availability

The processed MR data of the six volunteers used in this study are publicly available at https://doi.org/10.5281/zenodo.6563613 (accessed on 17 May 2022).

## Abbreviations

The following abbreviations are used in this manuscript:

[^18^F]FET	[^18^F]Fluoroethyl-L-tyrosine
ADC	Average diffusion coefficient
ASSD	Average symmetric surface distance
CCC	Lin’s concordance correlation coefficient
(D)CNN	(Deep) convolutional neural network
DICOM	Digital imaging and communications in medicine
DTI	Diffusion tensor imaging
DW	Diffusion-weighted
EPI	Echo planar imaging
GBM	Glioblastoma
IDH	Isocitrate dehydrogenase
MAE	Mean absolute error
MRE	Median relative error
MR(I)	Magnetic resonance (imaging)
MSE	Mean squared error
PDE	Partial differential equation
PET	Positron emission tomography
ReLU	Rectified linear unit
T1Gd	T1-weighted sequence with injection of gadolinium-based contrast agent
T2-FLAIR	T2-weighted sequence with fluid-attenuated inversion recovery

## Appendix A. FSL DTI Data Processing

The DICOM files of both acquisitions—with and without phase-encoding polarity inversion—were first converted into NIfTI format using dcm2nii.exe available in the MRIcroGL software (version 1.0.20180623) [55]. The two resulting series of four files with extensions .bvals, .bvecs, .json, and .nii were then renamed dti_pepolar_0 and dti_pepolar_1, for the acquisition with and without phase-encode polarity inversion, respectively. An acquisition parameter file acqparams.txt specifying for each acquisition the phase-encode direction and the total readout time was then created and is provided in Listing A1. For GE scanners, the total readout time $t_{\mathrm{read}}$ [s] is given by:

(A1) $$t_{\mathrm{read}}=\left(n_{y}\;f_{\mathrm{acc}}-1\right)s_{\mathrm{echo}}\times 10^{-6}$$

where $n_{y}$ is the acquisition matrix size along the phase-encode direction (DICOM tag (0018,1310)), $f_{\mathrm{acc}}$ is the total phase acceleration factor given by the product of the ASSET and ARC factors (DICOM tag (0043,1083), ASSET/ARC), and $s_{\mathrm{echo}}$ is the effective echo spacing (DICOM tag (0043,102C)) [μs].

The Linux bash script used for DTI data processing using FSL (version 5.0.9-4) [33] is provided in Listing A2. Lines 3–5 gather both b=0 volumes into a single 4D volume b0_merged.nii.gz. Line 7 executes topup on the merged volume with acquisition parameters in acqparams.txt, which generates files b0_topup.nii.gz (the corrected b=0 volumes), topup_results_fieldcoef.nii.gz (the estimated susceptibility field), and topup_results_movpar.txt (the estimated movement parameters). Line 9 computes the average of the two corrected volumes in b0_topup.nii.gz and stores it as b0_topup_mean.nii.gz. Line 10 computes a brain mask for b0_topup_mean.nii.gz using bet [56] and generates files b0_topup_brain_mask.nii.gz (the computed brain mask) and b0_topup_brain.nii.gz (the masked volume). Lines 12–14 create a file specifying for each 3D volume in the 4D volume dti_pepolar_0.nii (one 3D volume per diffusion direction) the corresponding acquisition parameter line in the acqparams.txt file. Line 16 runs eddy with the ‘replace outliers’ option --repol on dti_pepolar_0.nii, given the previously detailed files and generates various result files including the corrected 4D volume dti_pepolar_0_eddy.nii.gz. Line 8 finally runs dtifit with the ‘save tensor’ option --save_tensor on the distortion-corrected diffusion data dti_pepolar_0_eddy.nii.gz, which generates the fitted diffusion tensor file dti_pepolar_0_eddy_tensor.nii.gz and various tensor-derived data.

**Listing A1.** Acquisition parameter file provided to topup. The first three numbers of each line specify the phase-encode direction in the image coordinate system for the acquisition with (first line) and without (second line) phase-encode direction inversion. The last number of each line is the readout time $t_{\mathrm{read}}$ given by Equation (A1).

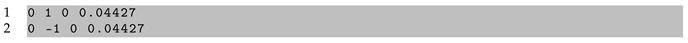

**Listing A2.** Linux bash script for DTI data processing using FSL.

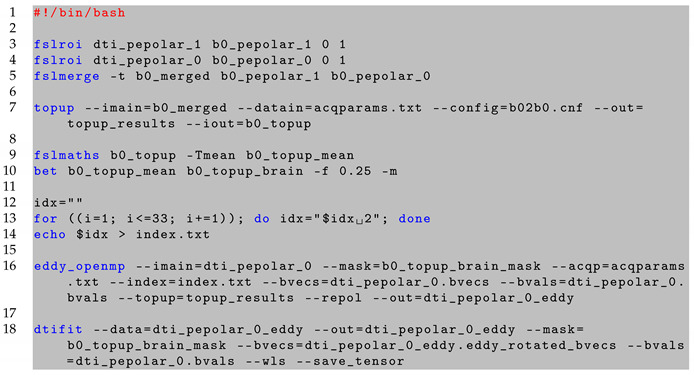

## Appendix B. Publicly Available Data

The processed MR data of the six healthy volunteers used for this study are publicly available at https://doi.org/10.5281/zenodo.6563613 (accessed on 17 May 2022). For each volunteer, a directory is provided containing the brain_domain.mha, unit_diffusion_tensor.mha, and unit_proliferation_rate.mha files in MetaImage format. brain_domain.mha contains the segmented brain map as derived in Section 2.3.4, stored as an unsigned short 3D image. Values 0, 2, 3, and 4 correspond to background, cerebrospinal fluid, gray matter, and white matter voxels, respectively. unit_diffusion_tensor.mha contains the six independent components $d_{xx}$, $d_{xy}$, $d_{xz}$, $d_{yy}$, $d_{yz}$, and $d_{zz}$ of the unit tumor cell diffusion tensor as derived in Section 2.3.6, stored as a double 4D image. unit_proliferation_rate.mha contains a dummy proliferation rate field stored as a double 3D image with value 1 for white and gray matter voxels and 0 elsewhere, to be scaled by the proliferation rate ρ and fed to the tgstkFiniteDifferenceReactionDiffusionTumourGrowthFilter (see https://cormarte.github.io/tgstk/html/classtgstk_finite_difference_reaction_diffusion_tumour_growth_filter.html, accessed on 17 May 2022).

## Appendix C. Additional Results

Additional examples of true and estimated tumor cell-density distributions from the test set for times $t_{2-4}$ obtained as described in Section 2.6 are depicted in Figure A1 and Figure A2, along with the corresponding absolute error maps and imaging contours.
